# Strength of Compressed Reinforced Concrete Elements Reinforced with CFRP at Different Load Application Eccentricity

**DOI:** 10.3390/polym15010026

**Published:** 2022-12-21

**Authors:** Petr P. Polskoy, Dmitry Mailyan, Alexey N. Beskopylny, Besarion Meskhi, Aleksandr V. Shilov, Artur Umarov

**Affiliations:** 1 Department of Reinforced Concrete Structures, Faculty of Industrial and Civil Engineering, Don State Technical University, Gagarin, 1, 344003 Rostov-on-Don, Russia; 2 Department of Transport Systems, Faculty of Roads and Transport Systems, Don State Technical University, 344003 Rostov-on-Don, Russia; 3 Department of Life Safety and Environmental Protection, Faculty of Life Safety and Environmental Engineering, Don State Technical University, 344003 Rostov-on-Don, Russia

**Keywords:** concrete, reinforced concrete, polymer composite materials, carbon fiber, compressed elements, columns, eccentricity, longitudinal force

## Abstract

Currently, many studies are devoted to the use of polymer composite materials to increase the strength and stability of concrete elements. In compressed reinforced concrete elements, the bearing capacity depends on the eccentricity of the external application of the external force and the corresponding stress-strain state, as well as the location and number of composite materials glued to the surface of the structure. The choice of a scheme for placing composite materials depending on the stress state of the structure is an urgent scientific problem. At the same time, the issue of central compression and the compression of columns with large eccentricities has been well studied. However, studies conducted in the range of average eccentricities often have conflicting results, which is the problem area of this study. The primary aim of this study was to increase the strength and stiffness of compressed reinforced concrete elements reinforced with composite materials, as well as a comparative analysis of the bearing capacity of ten different combinations of external longitudinal, transverse, and combined reinforcement. The results of testing 16 compressed columns under the action of various eccentricities of external load application (*e*_0_/*h* = 0; 0.16; 0.32) are presented. It is shown that the use of composite materials in strengthening structures increases the bearing capacity up to 41%, and the stiffness of the sections increases up to 30%. Based on the results of the study, recommendations are proposed for improving the calculation method for inflexible columns reinforced in the transverse direction, which take the work of concrete under the conditions of a three-dimensional stress state into consideration.

## 1. Introduction

An analysis of the modern construction market indicates that, along with the growth in capital construction, the volume of investments in the repair, restoration, and strengthening of building structures will certainly grow. In some cases, the volume of such investments is compared with the volume for new construction. In practice, the use of composite materials is increasing [1,2,3]. In construction, they are most widely used in the restoration [4,5,6] and strengthening of structures [7,8], which have insufficient bearing capacity. The use of composite materials in construction is justified by their high tensile strength, low weight, manufacturability of reinforcement, and linear relationship between stresses and strains up to their destruction.

Polymer composite materials are used in modern construction in various versions and technological forms. The use of polymer fibers as dispersed reinforcement is widely known [9,10,11,12]. This approach gives effective results at the stage of manufacturing structures and is rarely used in restoration or repair work.

Reinforcement with polymer composite rods, which are used instead of steel reinforcement, is widely used [13,14,15,16]. The use of polymer rods instead of steel ones is very attractive due to their lighter construction, high corrosion resistance, and significant bearing capacity. At the same time, the insufficient rigidity of glass or polypropylene polymer rods reduces the deformation characteristics of concrete in tension [17], which does not allow full use of their advantages. The use of basalt reinforcement [18] is also limited due to the sudden brittle fracture of the rods, which is a problem.

In recent years, polymer composite materials have been widely used as external reinforcement [19,20,21,22,23,24]. This technology for strengthening and repairing existing structural elements has shown effectiveness in various elements, including beams [25,26], columns [27,28,29], slabs [30,31], and others.

An experimental approach to bending reinforcement of reinforced concrete beams with reinforcement on the outer side using carbon fiber fabrics is presented in [32]. The research program included testing eight full-scale reinforced concrete beams for four-point bending to failure. The results of the experiment show that the reinforcement of the beams with CFRP laminates increased the first crack load and the ultimate load-bearing capacity of the beams by up to 141% and 174%, respectively, compared to the control beam.

External reinforcement of the beams due to the attachment of a polymer plate in the lower tension zone [33] leads to the delamination of the FRB plate and is one of the critical failure modes. The authors of [33] studied the effect of CFRP stirrups for reinforcing reinforcement and showed the effectiveness of this approach.

Bridge structures operating under conditions of cyclic dynamic loads were the object of study in [34]. Strengthening was carried out due to additional prestressing using polymer reinforcement by reinforcing the lower chord with a plate applied to the destroyed, worn-out bridge. The best stiffness values in the load range of 200–400 kN were shown by the prestressing method, which approximately doubled the stiffness.

An important structural element is the beam-to-column junction area, which experiences stress concentration. In [35], several parameters have been defined regarding the contribution of CFRP sheets attached externally to connection panels. The results of numerical and experimental studies have shown the efficiency from 25% to 87% of various variants for strengthening the structure.

The long-term performance of the laminate in the joint area was investigated in [36] and associated with the deterioration of the mechanical properties of the laminates in the environment. The study involved testing samples of a conventional new CFRP laminate in heated water at 23, 45, and 60 °C for 224 days. The CFRP tensile strength and modulus of elasticity decreased by a maximum of 33% and 26%, respectively, over 224 days of exposure. Based on the test data, an age-based long-term prediction model for the tensile strength of a CFRP laminate was developed.

In [37], the authors considered variants for strengthening structures with carbon fiber laminates in the cracking zone along the lower chord and developed a model for the cyclic strength of carbon fiber reinforcement under fatigue conditions.

The considered variants for strengthening structures are mainly aimed at increasing the tensile strength in the lower chord. At the same time, shear failure of reinforced concrete structures is a common phenomenon, and the use of polymer reinforcement, in this case, is not sufficiently described in the literature. In [38], the authors carried out an experimental study on RC of deep beams without shear reinforcement reinforced with fully wrapped FRP strips. The ratio of the cut span to the effective depth, the number of carbon layers, the span length between the carbon strips, and the type of FRP were chosen as study parameters. The results of the study showed an increase in shear strength from 38% to 120%, depending on the span between the FRP strips. The authors also note that there are no significant changes in the diagonal cracking loads of the RC-reinforced deep beams compared to the reference beams.

In recent years, the use of reinforced concrete columns in the construction of high-rise buildings and unique structures has expanded. Axial and eccentric loads lead to lateral deflections, which can critically affect the performance of the structure. In [39], the behavior of square, thin reinforced concrete columns reinforced with carbon fiber-reinforced polymer sheets was studied. Columns with a slenderness factor of 25 were studied and were loaded at eccentricity to cross-section ratios of 0, 0.24, 0.48, and 0.72. A method was proposed in which three longitudinal grooves were cut using a grinding machine, parallel to the longitudinal axis of the columns, which were filled with epoxy resin during strengthening.

The behavior of reinforced concrete columns made of plain concrete and fiber-reinforced concrete wrapped with CFRP sheets and load eccentricity factors $e_{0}/h$ = 0.29, 0.46, and 0.63 were fabricated and tested [40] under eccentric compressive load. The results showed that CFRP sheets improved the load-bearing capacity and ductility of reinforced concrete columns, while the presence of steel fibers in concrete had little effect on the load-bearing capacity of columns but made them more ductile.

The choice of the optimal scheme for strengthening the structure during repair and restoration is a scientific problem, especially with an eccentrically applied load [41]. Twenty-two reinforced concrete columns were reinforced using various patterns, including surface mounted CFRP strips in tension and compression zones, and tested eccentrically ($e_{0}/h$ = 0.38). The test results showed that the hybrid repair method significantly contributed to the restoration of the original load capacity with an average load increase of 16%. The type of destruction of the columns consisted mainly of the delamination and destruction of the concrete pavement in the compression zone, accompanied by the local buckling of the reinforcement. The hybrid technique has improved the deformability, toughness, and axial stiffness of the reinforced columns.

In [42], the results of an experimental study are presented, consisting of tests of nine rectangular reinforced concrete columns, including eight reinforced concrete columns limited by fiberglass, and one reinforced concrete column as a control sample tested in axial compression. The experimental program included an examination of the corner radius and FRP sheath thickness as key test variables. The corner radius ratio and FRP shell thickness are shown to have a significant effect on the stress-strain response of FRP-constrained concrete in rectangular columns.

The review shows that, with many studies, the issue and main gap of the effective use of longitudinal and transverse methods of strengthening compressed reinforced concrete elements remains unexplored, considering the actual stress-strain state, which depends on the eccentricity of the load application.

Thus, the purpose of this work was to study the numerical parameters of the efficiency of composite reinforcement variants depending on the variable parameters and to develop a methodology for their calculation.

The scientific novelty of the work lies in the experimental and analytical study of the assessment of the strength of compressed elements reinforced with composite materials at various eccentricity coefficients.

## 2. Materials and Methods

To study the features of the work of compressed reinforced concrete elements reinforced with composite materials, as well as to obtain data on their strength, a series of experiments was carried out.

At the first stage of the research program, 16 reinforced concrete columns were made. All samples had sections of 250 × 125 (*h*) mm and a length of 1200 mm.

Concrete on heavy aggregate was used in the manufacture. The strength of concrete in terms of compressive strength was determined from the results of testing concrete cubes with a rib size of 150 mm on a hydraulic press. The load on the samples increased at a constant rate of 0.05…0.06 MPa/cm^2^ per second until the moment of destruction. As a result, the compressive strength of concrete varied from 38.6 MPa to 50.6 MPa. Consumption of materials by weight for the preparation of 1 m^3^ of a concrete mixture is shown in Table 1.

The longitudinal reinforcement of the prototypes is made symmetrical and consists of 4 diameters of 12 mm with a tensile strength of 500 MPa (class A500). The percentage of reinforcement, in this case, was 1.44% to identify the maximum effect of concrete reinforcement with composite materials. 

The main transverse reinforcement is knitted in the form of closed collars with a diameter of 6 mm with a tensile strength of 500 MPa (class B500). Stirrups were installed in increments of 180 mm. In addition to the main transverse reinforcement, 6 structural meshes are installed at the ends of the columns§ with a step of 50 mm. Their purpose is to increase the strength of concrete within the length of the anchoring zone of the longitudinal working reinforcement of the columns. The grids are made with a mesh size of 50 × 40 mm from wire reinforcement with a diameter of 3 mm with a tensile strength of 500 MPa. The strength characteristics of the working reinforcement and stirrups were obtained from the results of tensile tests of 5 samples of each type. 

The tests were carried out on a GRM-20 universal tensile testing machine No. 1837 (Armavir, Russia) in accordance with GOST 12004-81. A strain gauge station measured longitudinal deformations based on 100 mm with a scale division of the indicators equal to 0.01 mm. The average test results of rod reinforcement of class A500 and B500 and wire with a diameter of 6.0 mm (used in meshes and clamps) are given in Table 2.

**Figure 1:**
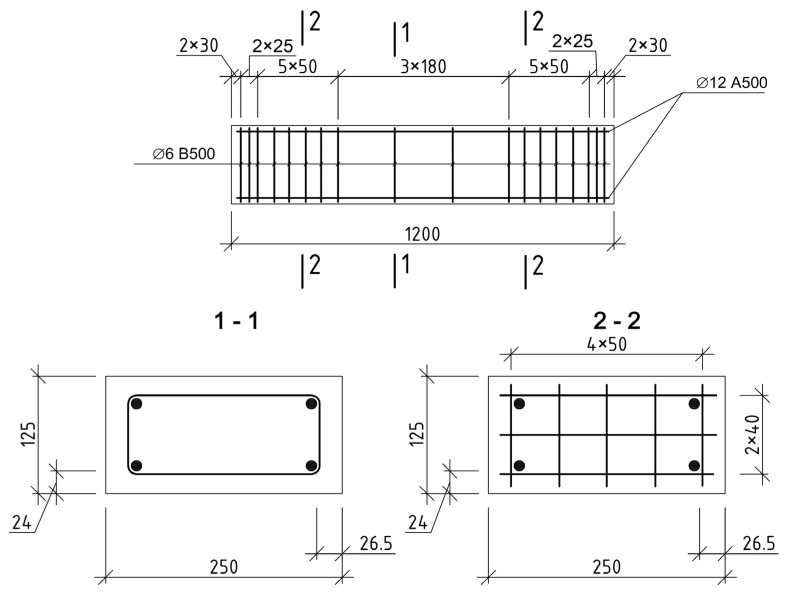
Design of frames of prototypes.

All columns were designed according to the recommendations [41]. The design of frames and grids is shown in Figure 1.

**Figure 2:**
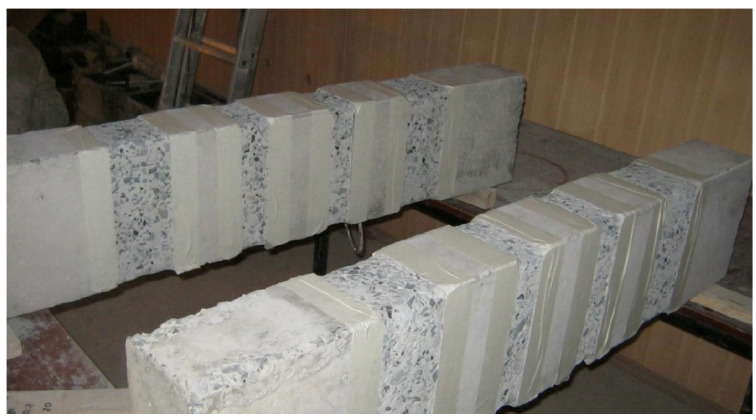
General view of the columns, prepared for reinforcement with stirrups and installation of end stirrups-anchors.

A general view of the sample prepared to reinforce the columns with transverse stirrups is shown in Figure 2.

**Figure 3:**
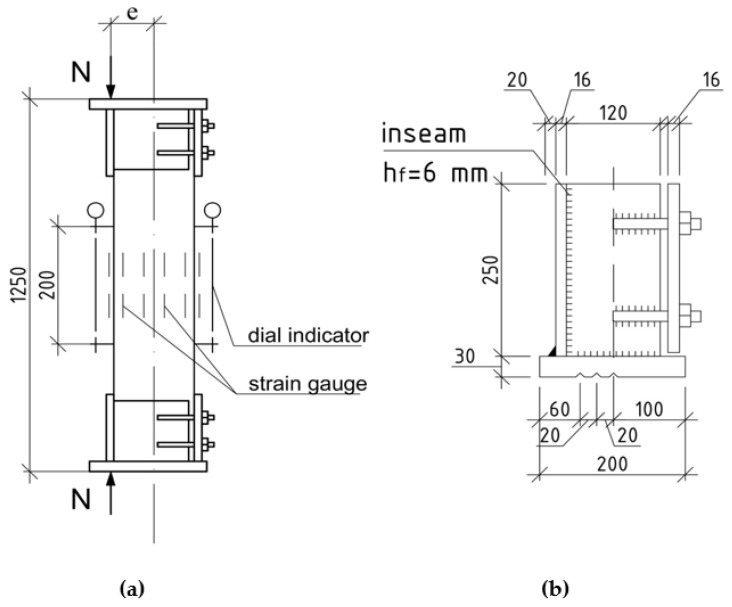
Head design: (**a**) is frontal view; (**b**) the device for eccentricity load.

To ensure three types of stress-strain state of the compressed elements, the load on the prototypes was transferred with a clearly fixed eccentricity through a specially designed steel head, which was bolted to the ends of the columns before testing. The design of the head is shown in Figure 3. This setup is designed to test samples for compression with eccentricity.

Three slots were made on the end plates of the indicated heads, located along the geometric axes of the prototypes and at distances of 2.0 and 4.0 cm from them. The load from the press was transferred to the columns through trihedral steel knives with a leg of 70 mm installed in these slots, thereby creating three types of stress-strain state. This is conditionally central compression at a value of $e_{0}/h$ = 0 (samples of series A); elements loaded at conditionally small eccentricities, when $e_{0}$ = 2.0 cm or $e_{0}/h$ = 0.16 (series B); loaded with large eccentricities at $e_{0}$ = 4.0 cm or $e_{0}/h$ = 0.32 (series C).

Three types of carbon fiber composite materials were used to reinforce the columns, namely carbon fabric, the density of which was 300 g/m^2^, carbon laminates (strips) that were 1.2 mm thick and 50 mm wide, and round rods Ø8 mm. All materials, including primer, putty, and adhesives, were provided by the Moscow branch of MBRACE–BASF BUILDING SYSTEMS LLC. Specifically, carbon laminates (MBRACE^®^LAM CF210/2400.50 × 1.4.100 m) were used as elements of the longitudinal composite reinforcement, and carbon fabric (MBRACE FIB CF 300/4900.300 g/5.100 m) was used as transverse reinforcement. The strength characteristics of carbon fabric are taken from the results of tests of three-layer samples. Average value of temporary resistance $\sigma_{fu}$ = 2888 MPa. The tensile strength of carbon laminates and round rods Ø8 mm is taken according to the data of the manufacturer’s certificate $\sigma_{fu}$ = 2400 MPa. The modulus of elasticity of the composite materials used in the study is 244 GPa.

**Figure 4:**
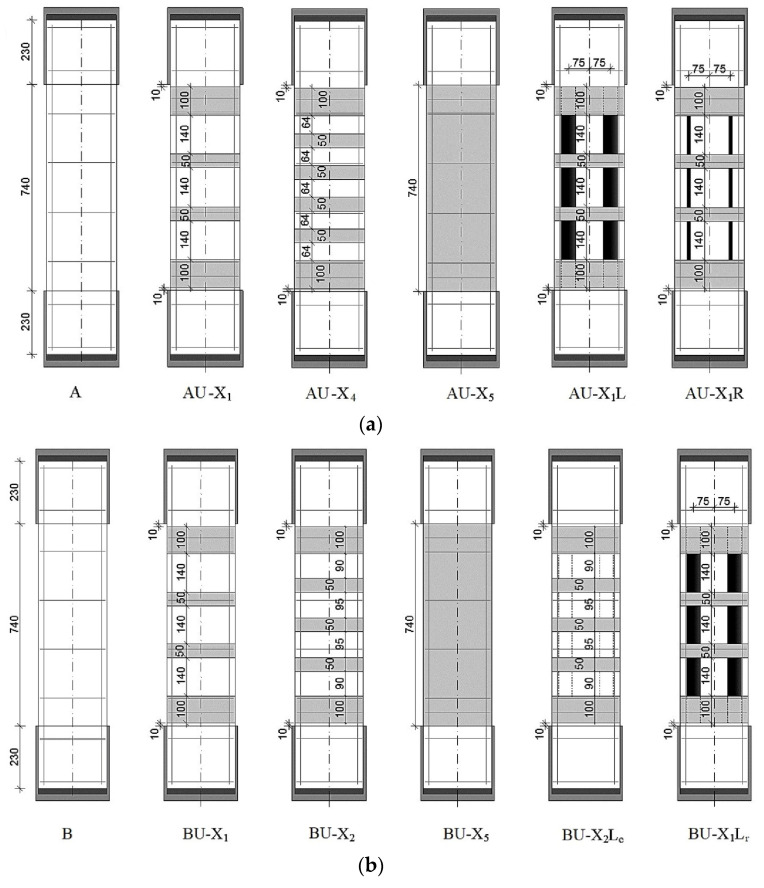

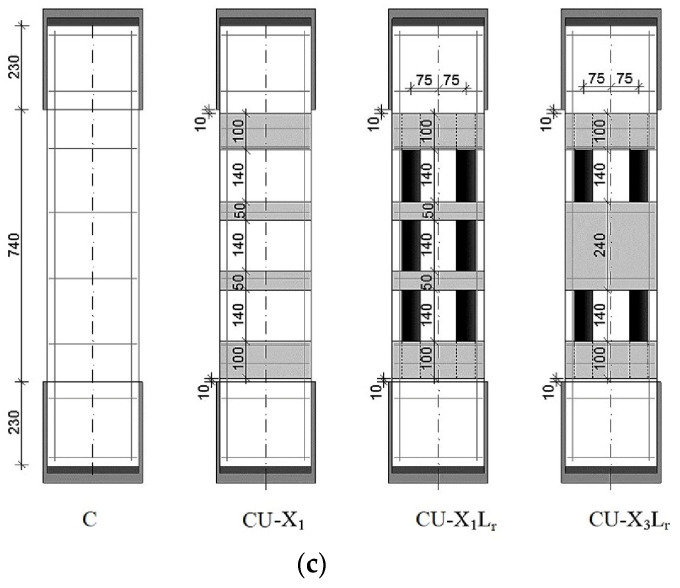
Variants for strengthening the columns with external transverse, longitudinal and combined composite reinforcement with an eccentricity of load application: (**a**) $e_{0}$ = 0; (**b**) $e_{0}$ = 2 cm; (**c**) $e_{0}$ = 4 cm.

Using these materials, 13 different reinforcement variants were developed: 5 variants for reinforcing samples of series A, 5 for series B, and 3 for series C. The reinforcement variants were divided into 3 types—external transverse, longitudinal, and combined reinforcement. The variants of reinforcements are shown in Figure 4. Each variant is indicated by an alphabetic and numeric code. It is presented as follows.

The first three capital letters of the alphabet—A; B; C—designate reference samples without reinforcement that were prepared for a constructive version of reinforcement with stirrups, which will be tested, respectively, with an axial eccentricity of the load application $e_{0}$ = 0; $e_{0}$ = 2.0 cm (0.16 h); $e_{0}$ = 4.0 cm (0.32 h).

The capital letter U, located next to the first three capital letters, indicates the presence of a reinforced prototype.

The capital letter X indicates the presence of one of the variants for external transverse reinforcement—a stirrup, and the number next to this letter is a variant of transverse reinforcement: (1)—stirrups with a width of $W_{f}$ = 50 mm located with a step in the axes $S_{f}$ = 190 mm (constructive reinforcement variant); (2)—stirrups 50 mm wide with a pitch of 145 mm; (3)—a stirrup with a width of $W_{f}$ = 240 mm, installed in the center of the column with a typical clearance to the stirrup-anchors; (4)—stirrups with a width of $W_{f}$ = 50 mm with a gap between the stirrups of 64 mm; (5)—three-layer stirrup-clip along the entire length of the columns

The capital letter *L* indicates the presence of external longitudinal reinforcement with laminates (strips) 50 mm wide and 1.2 mm thick.

The capital letter *R* indicates reinforcement with carbon-fiber rods Ø8 mm, glued symmetrically on both sides along the uprights into strips (grooves) cut in the protective layer with a depth of 20 mm.

The lowercase letters (c) and (r) indicate the presence of external longitudinal reinforcement, which is glued, respectively, from the side of the compressed (less compressed) face of the uprights, or the stretched one. The absence of lowercase letters (c) and (r) next to *L* or *R* means that flat or round composite reinforcements are glued symmetrically on both sides of the elements.

With external transverse reinforcement, the widths of the stirrups and the gaps between the transverse stirrups were varied. With external longitudinal reinforcement, the cross-sectional areas of composite materials and the variants for gluing reinforcement elements changed—symmetrically from two sides, or from one side, respectively, on a compressed (less compressed) or stretched face. All stirrups and a solid clip were made of three layers to ensure their sufficient rigidity, because according to studies [8], two-layer stirrups with a fabric density of 300 g/m^2^ did not provide adequate rigidity and were torn at the rounded corners of the prototypes.

Round longitudinal rods according to the AU-X1R variant were glued into longitudinal grooves—gaps, 20 mm deep, cut in the protective layer of concrete with a diamond saw along the reinforced elements. With combined reinforcement, the longitudinal reinforcement elements were glued first, followed by the transverse ones.

**Figure 5:**
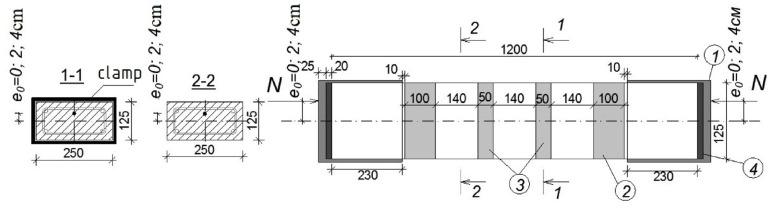
Structural version of the reinforcement of the columns with external transverse composite reinforcement and the load application scheme: 1—steel head, 2 and 3—end stirrup-anchor and ordinary reinforcement stirrups made of three layers of carbon fabric, 4—cement-sand mortar grade 200.

For all reinforcement variants, the prototypes, regardless of the presence of longitudinal reinforcement elements, had three-layer stirrups 100 mm wide, located next to the metal heads. They are called anchor stirrups—clips—and are designed to provide anchoring of the longitudinal elements of the composite reinforcement, i.e., putting them into work. The design of the reinforcement of the columns with external transverse composite reinforcement and the load application scheme are shown in Figure 5.

**Figure 6:**
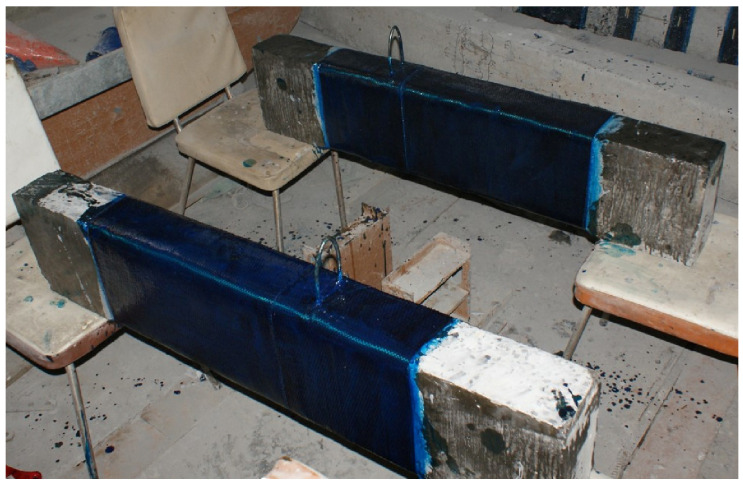
General view of the column, reinforced according to the X5 variant—a full clip of three layers of carbon fiber MBRACE FIB CF 300/4900.300 g/5.100 m.

A general view of the prototype reinforced according to the AU-X5 variant is shown in Figure 6.

The technique for strengthening prototypes was based on recommendations from the manufacturer of composite materials and algorithms for strengthening reinforced concrete structures, which are given in the annexes of regulatory documents of various countries on strengthening reinforced concrete structures with composite materials. The work sequence was adopted as follows:

- Surface marking according to reinforcement variants;
- Removal of cement-sand milk in the places of installation of reinforcement elements until large filler is exposed and corners are rounded with a radius of curvature 20 mm in places where stirrups or clips are glued;
- Dedusting the surface and applying a primer, if necessary—leveling the surface with an epoxy-based putty;
- Reinforcement of the structure according to the reinforcement variant.

After strengthening, the prototypes were kept until the moment of testing for 7 days until the adhesive composition was completely cured.

The strength characteristics of concrete for prototypes according to their reinforcement variants are shown in Table 3.

**Figure 7:**
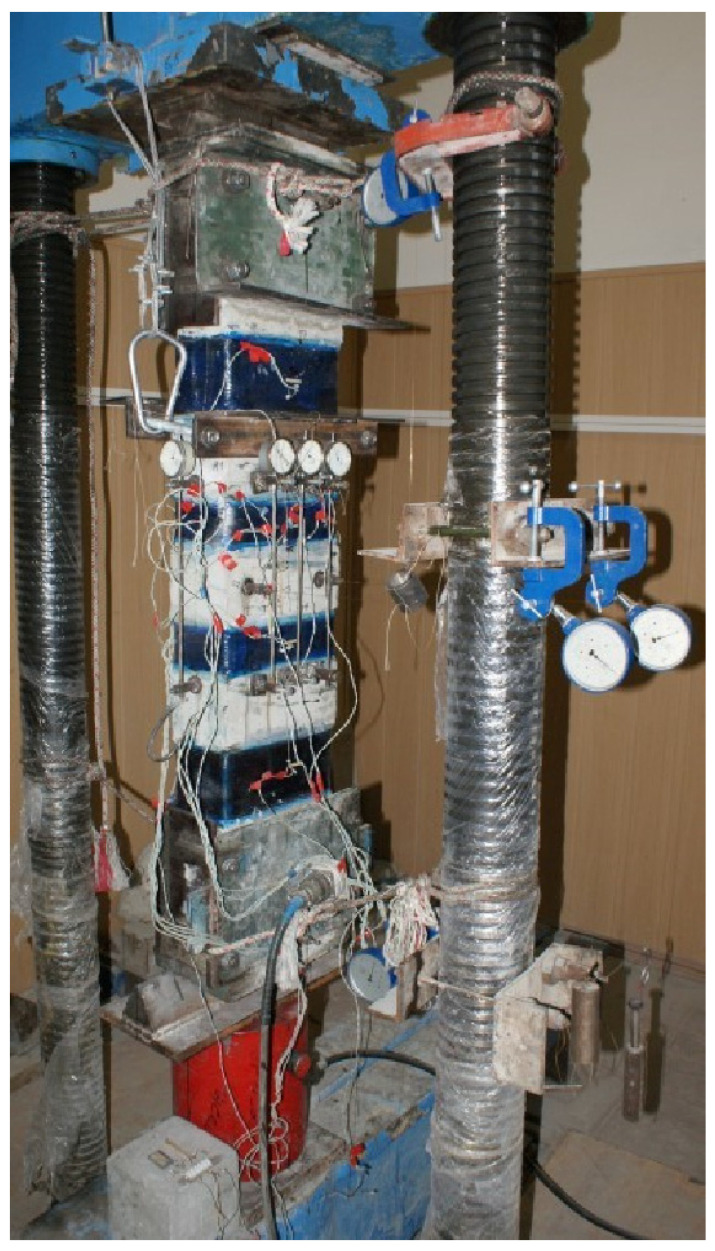
The design of the stand for testing columns with the location of mechanical devices and resistance strain gauges for measuring displacements and relative deformations.

Testing of prototypes was carried out on a specially equipped stand. Figure 7 shows a photograph of the prototype AU-X1, with the location of mechanical instruments and resistance strain gauges. To measure the displacements and relative deformations of concrete and composite materials, the following are installed on the sample: deflectometers of the Aristov system (at three points along the length of the columns), dial indicators with a division value of 0.01 mm (along four faces of the columns) to measure the average deformation of concrete along the length 300 mm, and resistance strain gauges with bases of 50 and 20 mm to measure the relative deformation of concrete and composite reinforcement, respectively. Recording of instrument readings was carried out visually or using strain gauge AID-4M.

Loading of prototypes was carried out using a DG-200 jack, developing a force of *N* = 2000 kN. The load on the columns was transmitted by two multidirectional forces through the blades of triangular isosceles knives. The latter were installed on a jack and under the top plate of the press. The force from the knives to the column was transmitted through steel heads with three slots to fix the eccentricities. Considering the thickness of the end plates of the heads, the estimated length of the columns was 1250 mm.

The loading of prototypes during the test was carried out with a step-increasing load with an intensity of 1/20 of the theoretical breaking load at levels up to $0.2N_{teor}$ and above $0.8N_{teor}$, and $0.1N_{teor}$ outside these limits. Holding under load at each stage of loading was 10–15 min, during which twice at the beginning and end of the stage, instrument readings were taken, and the width of cracks after their appearance was measured. Reference samples (without reinforcement elements) were tested first. The reinforced specimens were tested at the same load steps as the reference ones to be able to directly compare the test results and determine the effect of composite reinforcement on the change in the strength of the columns.

## 3. Results

In accordance with the program of experimental studies, all 16 samples were tested by short-term load to failure. Six columns were tested for central compression, six for relative eccentricity $e_{0}/h$ = 0.16, and four for eccentricity $e_{0}/h$ = 0.32.

The data obtained show that the effectiveness of external composite reinforcement is affected not only by the design of reinforcement variants but also by the stiffness of the reinforcement elements, as well as the value of the axial eccentricity of the load application $e_{0}$.

**Figure 8:**
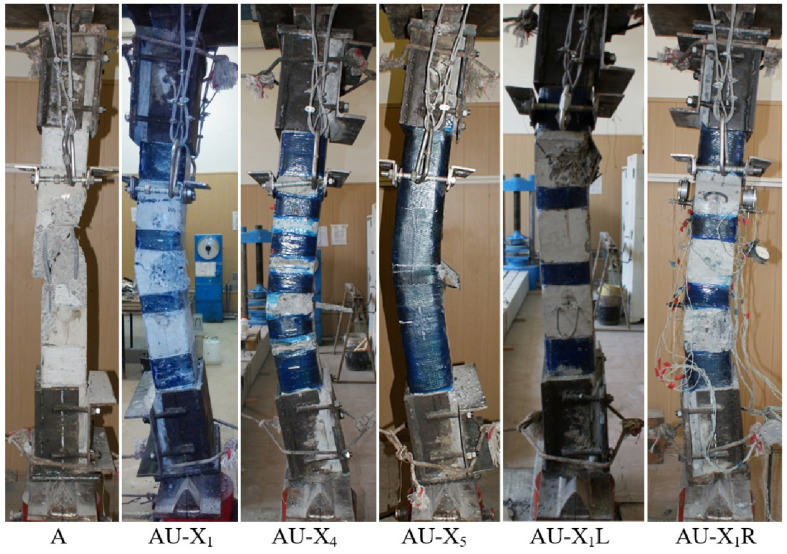
The nature of the destruction of the reference (A) and reinforced (AU) samples with an axial eccentricity of the load application *e*_0_ = 0.

**Figure 9:**
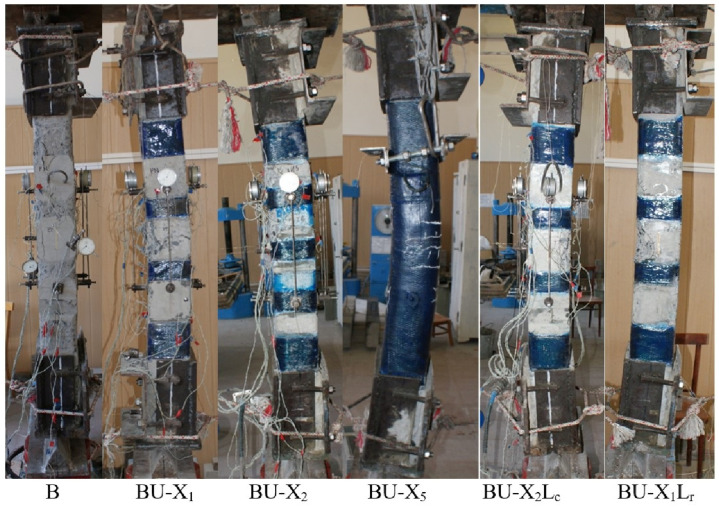
The nature of the destruction of the reference (B) and reinforced (BU) samples with an axial eccentricity of the load application *e*_0_ = 2 cm (*e*_0_ = 0.16 h).

**Figure 10:**
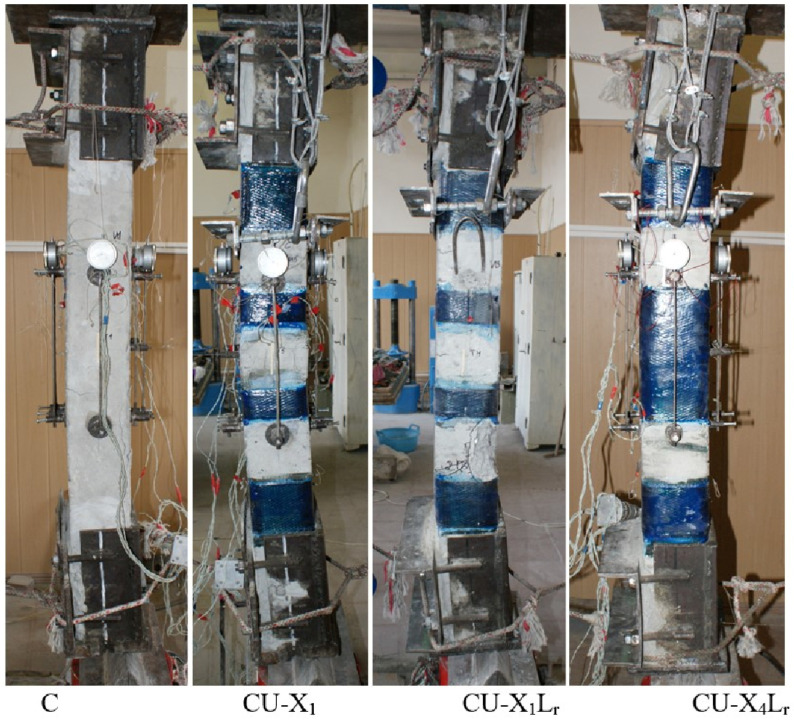
The nature of the destruction of the reference (C) and reinforced (CU) samples with an axial eccentricity of the load application *e*_0_ = 4 cm (*e*_0_ = 0.32 h).

The nature of the destruction of the samples (Figure 8, Figure 9 and Figure 10), as well as the graphs of the experimental values of deflections (Figures 12–14) and changes in relative deformations (Figures 15–17), allow us to note that the accepted values of the axial eccentricity of the load application *e*_0_ = 0; $e_{0}$ = 2 cm (0.16 h), $e_{0}$ = 4 cm (0.32 h), cover the entire spectrum of the main types of stress-strain state of compressed elements.

The influence of various reinforcement variants on the strength of reinforced samples is presented in Table 4, which shows the values of the coefficient of reinforcement $k_{f}$ equal to the ratio of the breaking load for reinforced samples to the same value of reference ones.

With eccentricity $e_{0}$ = 0, the greatest effect of amplification was shown by the X5 amplification variant in the form of a solid clip. The gain factor for this variant $k_{f}$ was 1.41 or 41%. 

The second largest coefficient $k_{f}$ was shown by the column (3), in which the gap between the composite reinforcement clamps $w_{f}$ = 50 mm wide was reduced to 65 mm, variant X4. The increase in strength, in contrast to the constructive version of the transverse reinforcement, was 39%.

Column (6), in which 4 carbon rods with a diameter of 8 mm were glued on both sides within the depth of the protective layer of concrete, showed an increase in strength of 20%.

**Figure 11:**
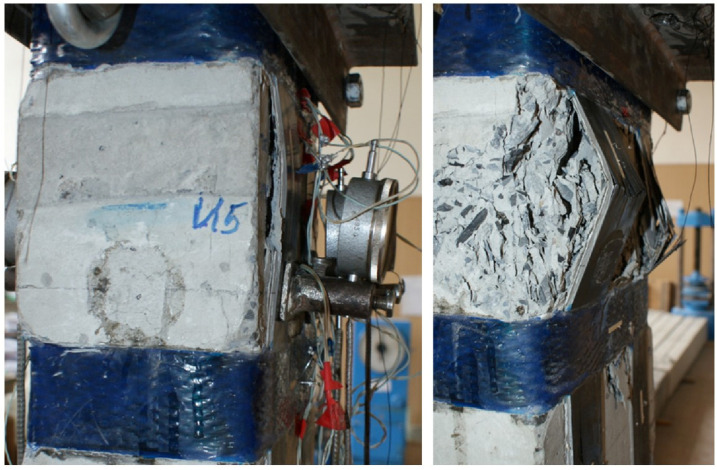
Local buckling of the lamellas during the test of the column (5) at a load level in the column of 1100 kN or 0.8*N_ult_* (**left**), at the moment of destruction (**right**).

Column (5), in which, simultaneously with the transverse clamps, two strips of carbon laminate were glued on opposite sides, also did not increase its strength [9]. This is quite understandable, since approximately at a load level of about 80%, the laminates were destroyed due to local buckling of the strips and their fracture (Figure 11).

Column (10), loaded with an eccentricity $e_{0}$ = 2.0 cm, also showed the maximum increase in strength, when reinforced with a continuous casing of the X5 type. The increase in strength was 42%, which is comparable to the increase in the strength of short legs under central loading.

Column (8), which was reinforced according to the X1 variant, as well as column (9) with X2 stirrups with a gap of 95 mm, and column (11), with a similar X2 clamp but in combination with two laminate strips glued to a more compressed edge, showed close strengths, which are 32–35% higher than the strength of the reference column (7).

Column (12) with X1 stirrups in combination with two strips only on the tension face showed a strength increment of 18%.

The prototypes loaded with an eccentricity $e_{0}$ = 4.0 cm showed that the degree of influence of the transverse reinforcement was less than the longitudinal one. This is explained by the fact that the clip effect for columns with different sign stresses is reduced due to the redistribution of compressive and tensile stresses within the section. Reducing the deformation of the tensile zone of concrete leads to an increase in the height of the compressed zone of concrete and an increase in the strength of normal sections.

**Figure 12:**
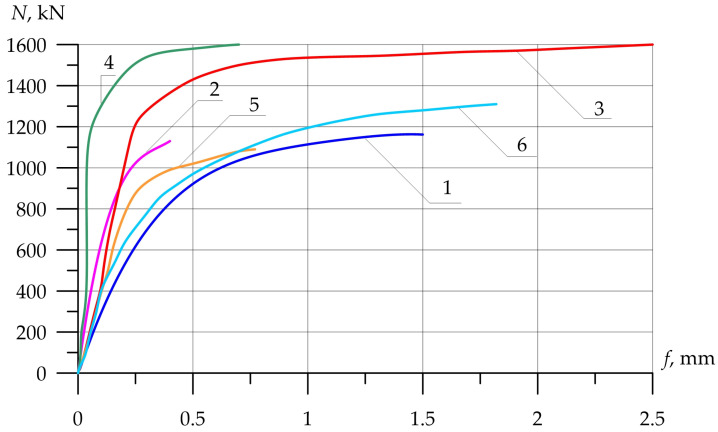
Comparison of experimental values of column deflections ($\lambda_{h}$ = 10) depending on the magnitude of the load and variants for composite reinforcement with axial eccentricity $e_{0}$ = 0. The numbers on the graphs correspond to the column numbers in Table 4.

**Figure 13:**
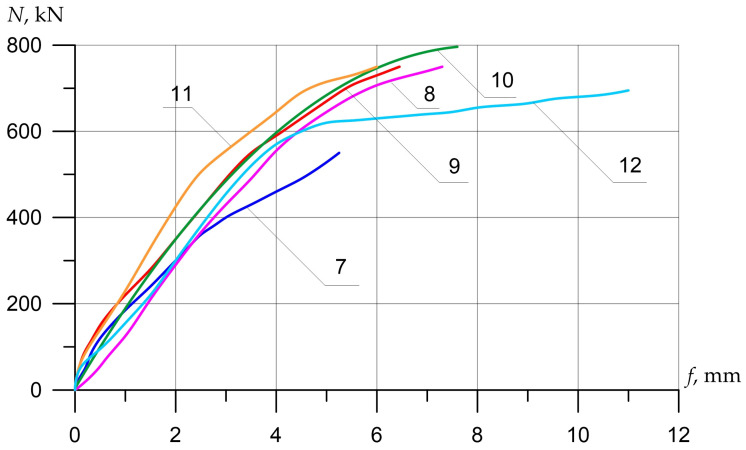
Comparison of experimental values of column deflections ($\lambda_{h}$ = 10) depending on the magnitude of the load and variants for composite reinforcement with axial eccentricity $e_{0}$ = 2.0 cm (0.16 h). The numbers on the graphs correspond to the column numbers in Table 4.

**Figure 14:**
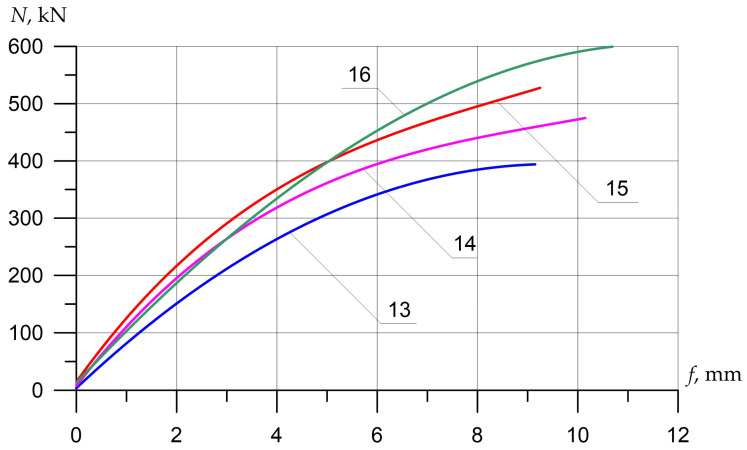
Comparison of experimental values of column deflections ($\lambda_{h}$ = 10) depending on the magnitude of the load and variants for composite reinforcement with axial eccentricity $e_{0}$ = 4.0 cm (0.32 h). The numbers on the graphs correspond to the column numbers in Table 4.

Columns (15) and (16), which were reinforced with external longitudinal composite reinforcement in combination with transverse stirrups, increased their strength compared to the reference (13) by 25 and 44%. Figure 12, Figure 13 and Figure 14 show the experimental dependences of the bearing capacity on the deflection of the columns. The numbers on the graphs correspond to the column numbers in Table 4.

It can be seen from Figure 12 that, compared with column (1), reinforced specimens (2) and (5) did not receive an increase in bearing capacity, but the deflections were significantly reduced. Samples reinforced according to schemes (3), (4), and (6) show an increase in bearing capacity up to 41%, and deflections reach 2.5 mm.

In Figure 13, the columns tested with eccentricity $e_{0}$ = 0.16 h show that reinforcement with composite materials, in all cases (8)–(11), gives an increase in bearing capacity of up to 40% compared to the control sample (7), and the ultimate deflections of the columns increase by 2.4 times.

Figure 14 shows columns tested with eccentricity $e_{0}$ = 0.32 h. The bearing capacity of samples (14)–(16), which were reinforced with composite materials, in all cases increases (up to 44%), while the deflections increase slightly (up to 17%), which is very important for columns experiencing a compressive force with a large eccentricity.

For compressed elements, with axial load application ($e_{0}$ = 0), the variant of external transverse reinforcement is more effective, especially when reinforced with a solid cage. For similar elements loaded at $e_{0}$ = 0.32 h (large eccentricities), on the contrary, the greatest effect is provided by the strengthening of the columns in the longitudinal direction.

## 4. Discussion

The presented results of the experiments performed showed an obvious effect on the bearing capacity and rigidity of the prototypes of all variable factors reflected in the research program variants for composite reinforcement and load application eccentricity.

Considering the presence of variation in the strength of concrete, further analysis was carried out using the reduced strength of $N_{red}$ prototypes. The specified value was obtained by multiplying the experimental strength of individual twin props $N_{i}$, by the reduction factor $k_{red}$, equal to the ratio of the strength of concrete of the reference and reinforced samples.

To obtain quantitative data on the effect of various reinforcement variants on the bearing capacity of the prototypes with a change in the eccentricity of the load application, additional processing of the experimental data was performed (Table 5).

Comparison of the ultimate reduced strength of the reinforced samples (column No. 7 of Table 3) shows that the maximum bearing capacity was shown by the samples reinforced with a solid three-layer casing according to the variant—X5 and tested at $e_{0}$ = 0.

The influence of the eccentricity, with its increase from 0 to 2.0 cm (0.16h) and especially at $e_{0}$ = 4.0 cm (0.32 h), turned out to be more significant; the efficiency of the continuous clip decreased by 2 and 2.5 times, respectively.

The variant of reinforcing the columns with wide stirrups at $w_{f}$ = 250 mm, located in the middle of the height of the flexible columns, together with clamps X1 (X2) showed an increase in strength up to 17% only at $e_{0}$ = 0. When changing the eccentricity $e_{0}$ to 2.0 and 4.0 cm, a noticeable increase in the bearing capacity of the columns with an increase the width of the clamps did not happen.

**Figure 15:**
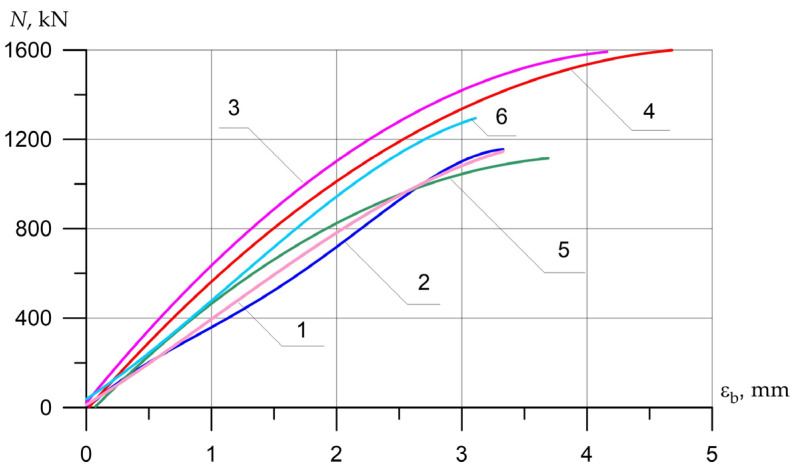
The average experimental values of the relative deformations of concrete in compression depending on the load and variants for composite reinforcement with axial eccentricity $e_{0}$ = 0. The numbers on the graphs correspond to the column numbers in Table 4.

**Figure 16:**
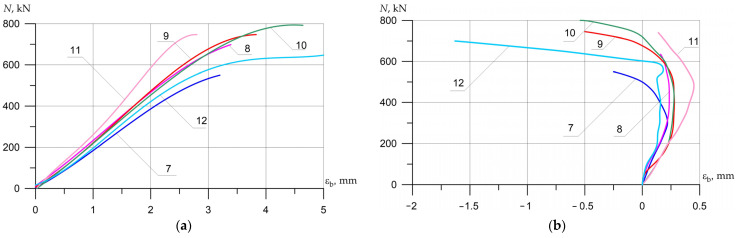
Comparison of the average experimental values of the relative deformations of concrete in compression (**a**) *ε_b_* and tension (**b**) $\varepsilon_{bt}$ on opposite faces of the columns ($\lambda_{h}$ = 10) depending on the load and variants for composite reinforcement with axial eccentricity $e_{0}$ = 2.0 cm (0.16 h). The numbers on the graphs correspond to the column numbers in Table 4.

**Figure 17:**
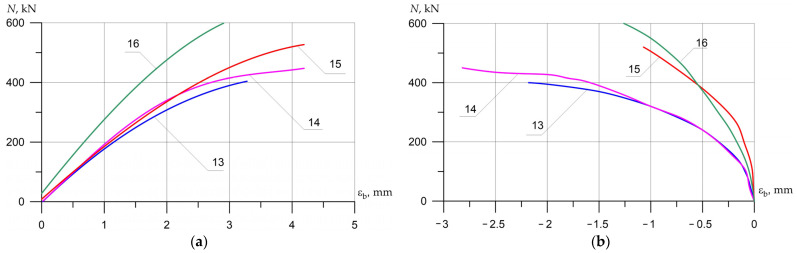
Comparison of the average experimental values of the relative deformations of concrete in compression (**a**) $\varepsilon_{b}$ and tension (**b**) $\varepsilon_{bt}$ on opposite faces of the columns ($\lambda_{h}$ = 10) depending on the load and variants for composite reinforcement with axial eccentricity $e_{0}$ = 4.0 cm (0.32 h).

Figure 15, Figure 16 and Figure 17 show the experimental dependences of the bearing capacity on the deflection of the columns. The numbers on the graphs correspond to the column numbers in Table 2.

With an eccentricity of $e_{0}$ = 2.0 cm (0.16 h), the X1 and X2 clamps in our experiments showed an increase in the load for the columns, comparable to the effect of a continuous clip.

The stirrups according to the X3 and X4 variants, which were used to strengthen the columns, were equal in width to one third of the length of the prototypes. They showed the effect of reinforcement only with complex reinforcement together with external longitudinal composite reinforcement. At the same time, with an increase in the width of the clamp, the influence of the clip effect decreased.

Finishing the assessment of the influence of external transverse reinforcement, it is also important to note that the effectiveness of the influence of transverse clamps glued to the longitudinal elements of external reinforcement decreases.

The longitudinal external reinforcement of the columns, using laminate at $e_{0}$ = 0 and 2.0 cm, did not affect their bearing capacity, since this reinforcement failed at a load in the range of (0.75…0.8)$N_{ult}$. With an eccentricity of $e_{0}$ = 4.0 cm, the laminates installed on the stretched face with the collar width $w_{f}$ = 50 mm showed a positive effect.

A clearer picture of the influence of reinforcement variants on the bearing capacity of prototypes can be traced when analyzing the experimental values of the composite reinforcement coefficient, which are obtained by dividing the reduced strength of the reinforced columns by the average strength of the reference samples. Their values are given in column 9 of the Table 3 and column 6 of Table 6.

**Figure 18:**
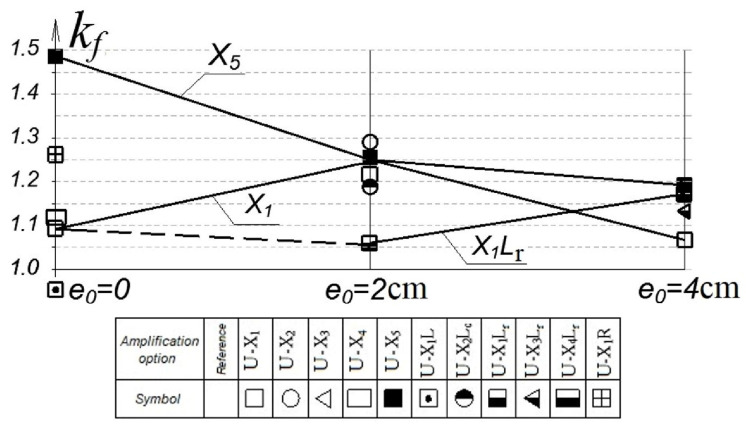
Influence of the load application eccentricity on the change in the composite gain *k_f_*.

The influence of the eccentricity of the load application on the change in the eccentricity of the load application on the change in the coefficient $k_{f}$ is shown in Figure 18.

The maximum bearing capacity was shown by columns reinforced with a solid three-layer casing according to the X5 variant, tested with an eccentricity $e_{0}$ = 0. At an eccentricity of $e_{0}$ = 2.0 cm (0.16 h), the efficiency of a continuous clip decreased by a factor of 2.

The second most effective variant was the X1R variant, in which the reinforcement was made by round rods with a diameter of 8 mm, glued into a groove cut in the protective layer of concrete. A similar picture was obtained in studies [10] where, instead of round rods, strips 25 mm wide and 1.4 mm thick were embedded in the groove.

At an eccentricity of $e_{0}$ = 2 cm (0.16 h), the efficiency of the solid clip decreased by a factor of 2 and became comparable with the combined longitudinal reinforcement of the stretched zone. At $e_{0}$ = 4 cm (0.32 h), the effect of the solid cage remained practically unchanged, while the value of the longitudinal reinforcement of the stretched zone, on the contrary, increased sharply and exceeded the efficiency of the solid cage for short columns.

The longitudinal external reinforcement of short studs using laminate at $e_{0}$ = 0 and 2.0 cm did not affect their load-bearing capacity, since this reinforcement failed at a load in the range of (0.75…0.8)$N_{ult}$. Similar results were obtained in [11].

With an eccentricity of $e_{0}$ = 4.0 cm, the laminates (strips) glued on the stretched edge, on the contrary, showed a positive effect. This is in good agreement with the provisions of [1,12].

The theoretical bearing capacity of columns under eccentric compression was determined by the following formula (1):

(1) $$N=\frac{R_{b3}\cdot b\cdot x\left(h_{0}-0,5x\right)+R_{sc}\cdot {A^{\prime }}_{s}\left(h_{0}-a^{\prime }\right)}{e}$$

where $R_{b3}$ is the strength of concrete under conditions of volume stress and is determined by formulas (2) and (3); *b* is the width of the section of the column; *x* is the height of the compressed zone of concrete in the section, determined by formulas (4) and (5); $h_{0}$ is the distance from the extreme compressed concrete fiber to the center of gravity of the least compressed or tensioned reinforcement; $R_{sc}$ is the compressive strength of the reinforcement; ${A^{\prime }}_{S}$ is the sectional area of the reinforcement located at the most compressed edge of the section; *a’* is the distance from the extreme compressed concrete fiber to the center of gravity of the reinforcement located at the most compressed edge of the section; *e* is the eccentricity of the external force application relative to the center of gravity of the least compressed or tensioned reinforcement in the section, determined by formula (2).

(2) $$R_{b3}=R_{b}+k_{ef}\cdot k_{e}\cdot R_{f}\cdot \mu_{f}$$

(3) $$k_{ef}=1-\frac{{\left(b-2\cdot r\right)}^{2}+{\left(h-2\cdot r\right)}^{2}}{2\cdot b\cdot h},k_{e}={\left(1-\frac{S_{w}}{2\cdot \left(\sqrt{h^{2}+b^{2}}-2\cdot r\right)}\right)}^{2},\;\mu_{f}=A_{f}/A$$

Depending on the eccentricity of the application of the external force, two cases of column failure were observed. In the first case, the stresses in the tensile reinforcement reached the yield point, and in the second case, the root cause of the destruction of the column was the achievement of stresses in the concrete of the compressed zone of the limit values. The calculation of the height of the compressed zone was carried out according to the following dependencies:

In the first case of calculation, when ξ=xh0≤ξR3

(4) $$x=\frac{N+R_{s}\cdot A_{s}-R_{sc}\cdot {A^{\prime }}_{s}}{R_{b3}\cdot b}$$

In the second case of calculation, when ξ=xh0>ξR3

(5) $$x=\frac{N+R_{s}\cdot A_{s}\cdot \left(1+\xi_{R3}\right)/\left(1-\xi_{R3}\right)-R_{sc}\cdot {A^{\prime }}_{s}}{R_{b3}\cdot b+2\cdot R_{s}\cdot A_{s}/\left(h_{0}\cdot \left(1-\xi_{R3}\right)\right)}$$

In both cases, the value of the boundary value of the height of the compressed zone $\xi_{R}$ was determined by the formula:

(6) $$\xi_{R}=\frac{x_{R}}{h_{0}}=\frac{0.8}{1+\varepsilon_{s.el}/\varepsilon_{b3}}$$

here $\varepsilon_{s,el}=R_{S}/E_{S}$ is relative deformation of tensile reinforcement at stresses equal to *R*_S_;

(7) $$\varepsilon_{b3}^{}=\varepsilon_{b2}+k_{f1}\cdot 2\cdot \mu_{f}\cdot \frac{R_{f,n}}{E_{b}},\;k_{f1}=1.25\times k_{e}-0.5$$

The calculated eccentricity of the application of the external force was determined by the formula (8)

(8) $$e=e_{0}\times \eta ,\;\eta =\frac{1}{1-N/N_{cr}},\;N_{cr}=\frac{\pi^{2}D}{l_{0}^{2}},\;D=k_{b}E_{b}I+k_{S}E_{S}I_{S},\;k_{b}^{}=\frac{0.15}{\phi_{l}(0.3+\delta_{e})}$$

where $e_{0}$ is the initial eccentricity of the external force application relative to the center of gravity of the least compressed or tensioned reinforcement in the section; *N* is the bearing capacity of the column; $N_{cr}$ is the conditional critical force, *D* is section stiffness.

Using the flexibility of the element, the value of the relative eccentricity, as well as the value of deflections, depending on the value of the coefficient *k*_e_ calculated by the formula indicated in the block diagram, the numerical value of the coefficient *k*_f2_ is determined, which is presented in the form of expression (9).

(9) $$\begin{array}{l} k_{f_{2}}^{theor}=k_{e}\left[\left(91.8-4.681\times \lambda_{h}\right)\times {\left(e_{0}/h\right)}^{2}+\left(1.581\times \lambda_{h}-40.115\right)\times \left(e_{0}/h\right)+0.0269\times \lambda_{h}+2.87\right]+\\ \;+\left(2.809\times \lambda_{h}-48.686\right)\times {\left(e_{0}/h\right)}^{2}+\left(20.312-0.982\times \lambda_{h}\right)\times \left(e_{0}/h\right)-0.0168\times \lambda_{h}+0.0663 \end{array}$$

Using the proposed algorithm, the theoretical bearing capacity of transversely reinforced posts was calculated, considering the proposed coefficients kf1, which corrects the ultimate relative deformation of concrete, and the coefficient *k*_f2_, which takes into account the effect of transverse reinforcement on the rigidity of structures. The calculation results are presented in Table 7.

The analysis of the obtained results showed that the introduction of the proposed coefficients kf1 and kf2 into the calculation ensures good convergence of the experimental and theoretical strength of the samples. The standard deviation of the experimental from the theoretical bearing capacity is 0.06.

The final calculation formula for determining the strength of concrete under conditions of volumetric stress state is:

(10) $$R_{b3}=R_{b}+k_{ef}\cdot k_{e}\cdot R_{f}\cdot \mu_{f}$$

(11) $$k_{ef}=1-\frac{{\left(b-2\cdot r\right)}^{2}+{\left(h-2\cdot r\right)}^{2}}{2\cdot b\cdot h},\;k_{e}={\left(1-\frac{S_{w}}{2\cdot \left(\sqrt{h^{2}+b^{2}}-2\cdot r\right)}\right)}^{2},\mu_{f}=A_{f}/A$$

Comparison of the experimental and theoretical values of the strength of the posts when calculating the bearing capacity of reinforced specimens using the formulas (1)–(11) proposed by the authors makes it possible to improve the convergence of the results of calculation and experiments, which positively affects the reliability of the proposed recommendations.

## 5. Conclusions

Tests of compressed reinforced concrete elements reinforced with various types of transverse, longitudinal, and combined composite reinforcement and tests at three values of the stress-strain state made it possible to obtain new data on the strength, deformability, and stiffness of reinforced samples, which indicate the high efficiency of composite strengthening of reinforced concrete structures. Based on the test results presented in this article, the following conclusions are drawn:

1. In centrally compressed columns, the maximum effect of increasing the bearing capacity is achieved with continuous wrapping with composite materials (41%). The use of external linear reinforcement elements located along the axis of action of the force does not give a positive result, since at a load level of about 80% of the limit, the laminates were destroyed due to local buckling of the strips and their fracture. The use of carbon-fiber rod reinforcement, glued symmetrically into the cut longitudinal grooves, gives the effect of increasing the bearing capacity up to 20% due to the guaranteed inclusion in work before the application of the breaking load.
2. In columns with an eccentricity value of e_0_ = 0.16 h, under conditions of nonuniform compression of the section, all options for strengthening in the transverse direction had a positive effect (from 30% to 40%). The use of external linear reinforcement elements located along the axis of action of the force from the side of the most compressed concrete fibers does not increase the bearing capacity, and the sticking of longitudinal laminates on the least compressed edge gives a minimal increase in strength due to the redistribution of forces in the cross-section through the transverse clamps.
3. In columns with an eccentricity of application of the external force e_0_ = 0.32 h, reinforcement in the transverse direction gives a minimal effect of increasing the bearing capacity (15%), and the use of longitudinal laminates on the side of tensioned concrete in combination with transverse clamps increases the strength up to 25%. Under the condition of the use of a wide collar in the middle part, which ensures guaranteed transmission of tensile stresses to the composite material, the strength increases up to 44%.

This study identified cases of possible effective use of longitudinally located reinforcing elements made of composite materials in compressed columns. The amount of FRP reinforcement and the configuration of the reinforcement, depending on the flexibility of the columns (the ratio of the length to the height of the section), requires further study.

## Figures and Tables

**Table 1:** Materials consumption for concrete preparation.

N	Concrete Type	Consumption of Materials by Weight per 1 m^3^ of Concrete						Density, *γ* kg/m^3^
		C	S		CS	W		
1	Heavy concrete	454	469		1290	180		2360

Designation: C is cement, S is sand, CS is crushed stone, W is water.

**Table 2:** Strength and deformation characteristics of steel bar reinforcement used for short columns.

Steel Class	Nominal Diameter, mm	$A_{S}$, cm^2^	$\sigma_{u}$, MPa	$\sigma_{Y}$, MPa
A500	12.0	1.313	612.7	530.8
B500	6.0	0.283	607.8	497

Designations: $A_{S}$ is a cross-section of the rebar in cm^2^; $\sigma_{u}$ is ultimate strength, MPa; $\sigma_{Y}$ is yield strength, MPa.

**Table 3:** Reinforcement variants and concrete strength characteristics of prototypes.

Column Series	Age of Concrete, Days	Column Code	Experimental Strength of Concrete, MPa			$E_{b}\times 10^{-3}$, MPa
			${\bar{R}}^{\exp}$	*B*	$R_{b}^{\exp}$	
2	3	4	5	6	7	8
A $e_{0}=0$	454	A	42.6	33.2	31.0	36,660
	546	AU-X_1_	38.6	30.0	28.2	35,580
	465	AU-X_4_	50.6	35.3	36.3	38,120
	416	AU-X_5_	38.7	30.1	28.3	35,600
	530	AU-X_1_L	40.8	31.8	29.8	36,250
	516	AU-X_1_R	38.7	30.1	28.3	35,600
B $e_{0}=2\;\mathrm{cm}\;\left(0.16\;h\right)$	523	B	38.9	30.3	28.4	35,670
	546	BU-X_1_	38.6	30.0	28.2	35,580
	523	BU-X_2_	38.9	30.3	28.4	35,670
	536	BU-X_5_	41.6	32.4	30.3	36,320
	536	BU-X_2_L_c_	41.6	32.4	30.3	36,320
	530	BU-X_1_L_r_	40.8	31.8	29.8	36,250
C $e_{0}=4\;\mathrm{cm}\left(0.32\;h\right)$	454	C	42.6	33.2	31.0	36,660
	454	CU-X_1_	42.6	33.2	31.0	36,660
	454	CU-X_1_Lr	42.6	33.2	31.0	36,660
	465	CU-X_3_Lr	50.6	39.4	36.4	38,120

Notes: In columns 5, 6 and 7, the following letter designations are accepted: ${\bar{R}}^{\exp}$ is the average value of the cubic strength of concrete; *B* is concrete compressive strength class and $R_{b}^{\exp}$ is experimental value of concrete axial compressive strength.

**Table 4:** Test results of reference and reinforced columns ($\lambda_{h}$ = 10) with axial eccentricity of load application $e_{0}$ = 0; 2.0 and 4.0 cm.

Characteristics of Prototypes	Number	Columns Code	Concrete Class *B*	Specification of Reinforcement Variants	Ultimate Strains		Experimental Values		$\begin{array}{c} \mathrm{Coefficient}\;\mathrm{of}\;\mathrm{Reinforcement}\\ k_{f}=N_{s,f}/N_{s} \end{array}$
					$\varepsilon_{b1}\times 10^{-3}$	$\varepsilon_{b2}\times 10^{-3}$	$\mathrm{Force},\;\mathrm{kN}\;N_{s};N_{s,f}$	$f_{}^{\exp}$ mm	
1	2	3	4	5	6	7	8	9	10
Series A. Axial eccentricity e_0_ = 0. Section 250 × 125 (*h*) mm *l*_0_ = 1200 mm, λ*_h_* = 10. Longitudinal reinforcement 4Ø12A500 (µs = 1.45) collars Ø6B500, s = 180 mm	1	A	33.2	Control sample	2.6	3.63	1150	1.165	1.0
	2	AU-X_1_	30.0	$\begin{array}{l} b_{f}=50\;\mathrm{mm};\\ s_{f}=190\;\mathrm{mm};s=140\;\mathrm{mm} \end{array}$	2.4	3.3	1190.5	0.4	1.035
	3	AU-X_4_	39.3	$\begin{array}{l} b_{f}=50\;\mathrm{mm};\\ s_{f}=115\;\mathrm{mm};s=65\;\mathrm{mm} \end{array}$	2.1	4.17	1600	2.52	1.39
	4	AU-X_5_	30.1	$b_{f}=720\;\mathrm{mm}$ (Clip along the entire length)	3.6	4.75	1625	0.7	1.41
	5	AU-X_1_L	31.8	$\begin{array}{l} b_{f}=50\;\mathrm{mm};s_{f}=190\;\mathrm{mm};\\ s=140\;\mathrm{mm};4L_{\mu } \end{array}$	2.4	3.74	1100	0.79	0.96
	6	AU-X_1_R	30.1	$\begin{array}{l} b_{f}=50\;\mathrm{mm};\;s_{f}=190\;\mathrm{mm};\\ s=140\;\mathrm{mm};\;4Ø8R \end{array}$	3.9	3.1	1379	1.82	1.199
Series B. Axial eccentricity e_0_ = 2 cm. Section-250 × 125 (*h*) mm l_0_ = 1200 mm, λ_h_ = 10. Longitudinal reinforcement 4Ø12A500 (µs = 1.45) collars Ø6B500, s = 180 mm	7	B	30.3	Control specimen	−0.25	3.25	592.5	5.2	-
	8	BU-X_1_	30.0	$\begin{array}{l} b_{f}=50\;\mathrm{mm};\\ s_{f}=190\;\mathrm{mm};s=140\;\mathrm{mm} \end{array}$	0.05	3.4	778.9	7.28	1.315
	9	BU-X_2_	30.3	$\begin{array}{l} b_{f}=50\;\mathrm{mm};\\ s_{f}=145\;\mathrm{mm};s=95\;\mathrm{mm} \end{array}$	−0.51	3.8	794.7	6.5	1.34
	10	BU- X_5_	32.4	$b_{f}=720\;\mathrm{mm}$ (Clip along the entire length)	−0.5	4.63	844.0	7.6	1.42
	11	BU- X_2_Lc	32.4	$\begin{array}{l} b_{f}=50\;\mathrm{mm};\\ s_{f}=145\;\mathrm{mm};s=95\;\mathrm{mm} \end{array}$ 2 carbon laminate in the compressed zone: $b=50\;\mathrm{mm};t_{}=1.4\;\mathrm{mm}$	−0.01	2.75	800.0	6.07	1.35
	12	BU-X_1_Lr	31.8	$\begin{array}{l} b_{f}=50\;\mathrm{mm};\\ s_{f}=190\;\mathrm{mm};s=140\;\mathrm{mm} \end{array}$ 2 carbon laminate in the tensile zone: b = 50 mm, t =1.4 mm	−1.6	5.0	700.0	11.3	1.18
Series C. Axial eccentricity e_0_ = 4 cm. Section-250 × 125 (*h*) mm l_0_ = 1200 mm, λ_h_ = 10. Longitudinal reinforcement 4Ø12A500 (µs = 1.45) stirrups Ø6B500, s_w_ = 180 mm	13	C	33.2	Control element	−2.2	3.3	422.2	9.15	-
	14	CU-X_1_	33.2	$\begin{array}{l} b_{f}=50\;\mathrm{mm};\\ s_{f}=190\;\mathrm{mm};s=140\;\mathrm{mm} \end{array}$	−2.7	4.23	482.5	10.3	1.148
	15	CU-X_1_Lr	33.2	$\begin{array}{l} b_{f}=50\;\mathrm{mm};\\ s_{f}=145\;\mathrm{mm};s=95\;\mathrm{mm} \end{array}$ 2 carbon laminate in the tensile zone: b=50 mm;t=1.4 mm	−1.1	4.1	530	9.2	1.25
	16	CU-X_3_Lr	39.4	$b_{f}=50\;\mathrm{mm};s_{f}=190\;\mathrm{mm};$ Stirrup in center $b_{f}=240\;\mathrm{mm}$ 2- carbon laminate in the tensile zone: b=50 mm;t=1.4 mm	−1.25	2.95	608	10.87	1.44

**Table 5:** Comparison of the reduced experimental strength of reference and reinforced columns when changing variants for external composite reinforcement.

Num.	CONCRETE Class *B*, MPa	Experimental Force (kN) at:		Normalized Force (kN) at:				$\mathrm{Reduced}\;\mathrm{Load}\;\mathrm{Increment}\;(\mathrm{kN})\;\Delta N_{s,f}^{red}$	$\mathrm{Rate}\;\frac{N_{\Delta ,}{}_{ult}}{N_{s,f}}$	$\mathrm{Coefficient}\;N_{f}^{red}/{\bar{N}}_{s}^{red}$	
		$\begin{array}{l} \mathrm{Collapse}\;N_{s},\\ N_{sf}^{} \end{array}$	$\mathrm{Ultimate}\;\mathrm{Strain}\;N_{\Delta ,}{}_{ult}$	$\mathrm{Collapse}N_{s}^{};N_{sf}^{}$		$\mathrm{Ultimate}\;\mathrm{Strain}\;N_{\Delta ,}{}_{ult}$					
										$\mathrm{Experiment}\;k_{s,f}^{red}=\frac{N_{f}^{red}}{{\bar{N}}_{s}^{red}}$	$\mathrm{At}\;\mathrm{Ultimate}\;\mathrm{Strain}\;k_{\Delta ,ult}^{red}=\frac{N_{\Delta ,ult}^{red}}{{\bar{N}}_{\Delta ,ult}^{red}}$
1	2	3	4	5		6		7	8	9	10
1	33.2	1150	1150.0	1150.0		1150.0		-	1.0	1.0	1.0
2 *	30.0	1190.5	1190.5	1317.5		1317.5		167.5	1.0	1.093	1.093
5 *	31.8	1100.0	1100.0	1148.4		1148.4		-	1.0	1.0	1.0
					1205.3		1205.3				
3	39.3	1600.0	1600.0	1351.6		1351.6		146.3	1.0	1.12	1.12
4	30.1	1625.0	1625.0	1792.3		1792.3		587.0	1.0	1.487	1.487
6	30.1	1379.0	1379.0	1521.0		1521.0		315.7	1.0	1.262	1.262
7	30.3	592.5	550.0	592.5		550.0		-	0.92	1.0	1.0
12 *	31.8	700.0	630.0	667.06		600.3		37.2	0.9	1.06	1.05
					629.7		572.6				
8	30.0	778.9	707	786.7		714.0		157.0	0.91	1.249	1.24
2	4	5	6	7		8		9	10	11	12
9	30.3	794.7	730.0	794.7		730.0		165.0	0.92	1.262	1.27
10	32.4	844.0	750.0	789.3		701.4		159.6	0.89	1.253	1.225
11	32.4	800.0	750.0	748.1		701.4		118.4	0.94	1.188	1.22
13	33.2	422.2	335.0	422.2		335.0		-	0.794	1.0	1.0
14 *	33.2	482.5	391.0	482.5 452.3		391.0 357.5		330.2	0.788	1.067	1.063
15	33.2	530.0	438.0	530.0		438.0		77.7	0.826	1.172	1.225
16	39.4	608.0	453.0	512.3		381.7		60.0	0.745	1.133	1.07

Notes: (1) Columns marked with (*) are transferred to the number of reference ones, because showed a small increase in strength comparable to the spread of the cubic strength of concrete. (2) Reduced strength of reinforced columns $N_{s,f}^{red}$ was obtained by multiplying the experimental strength by a reduction factor equal to the ratio of concrete classes of the reference and reinforced samples $k^{red}=B_{s}/B_{sf}$. The denominator of column 6 shows the average strength value for the reference and marked (*) prototypes ${\bar{N}}_{s}^{red}$; (4) The code of the columns is given in Table 2.

**Table 6:** Experimental values of the composite gain ($k_{f}^{red}$ ) when comparing the reduced strength of the columns $N_{s.f}^{red}$.

Specimens’ Series	Num	Column Code	Concrete Class B, MPa	$\mathrm{Reduced}\;\mathrm{Load}\;\mathrm{Capacity}\;N_{s}^{red};N_{sf}^{red}$	$\mathrm{Coefficient}\;\mathrm{of}\;\mathrm{Reinforcement}k_{f}^{red}=N_{sf}^{red}/N_{s}^{red}$	$\mathrm{Coef}.\;\mathrm{Buckling}\;\phi_{s}^{};\phi_{sf}^{}$	$\phi_{sf}^{}/\phi_{s}^{};$
1	2	3	4	5	6	7	8
Short Columns							
Serie A. Axial Eccentricity $e_{0}$ = 0.	1	A *	33.2	1205.3	1.0 *	1.0	1.0
	2	AU-X_1_	30.0	1317.5	1.093 *	1.12	1.12
	3	AU-X_4_	39.3	1351.6	1.121	1.216	1.216
	4	AU-X_5_	30.1	1792.3	1.487	1.523	1.523
	5	AU-X_1_L	31.8	1148.4	0.953 *	0.99	0.99
	6	AU-X_1_R	30.1	1521.0	1.262	1.295	1.295
Serie B. Axial Eccentricity $e_{0}$ = 2.0 cm (0.16 h)	7	B *	30.3	629.7	1.0 *	-	-
	8	BU-X_1_	30.0	786.7	1.249	-	-
	9	BU-X_2_	30.3	794.7	1.262	-	-
	10	BU-X_5_	32.4	789.3	1.253	-	-
	11	BU-X_2_L_c_	32.4	748.1	1.188	-	-
	12	BU-X_1_L_r_	31.8	667.0	1.06 *	-	-
Serie C. Axial Eccentricity $e_{0}$ = 4.0 cm (0.32 h)	13	C *	33.2	452.3	1.0 *	-	-
	14	CU-X_1_	33.2	482.5	1.067 *	-	-
	15	CU-X_1_L_r_	33.2	530.0	1.172	-	-
	16	CU-X_3_L_r_	39.4	512.3	1.133	-	-

Note: An asterisk (*) marks the samples, which were included in the number of reference samples during further processing of the results.

**Table 7:** Comparison of experimental and theoretical strength values of posts reinforced in the transverse direction with composite materials, considering the proposed coefficients *k*_f1_ and *k*_f2_.

Sample’s Code	Parameters		Experimental Results		Calculation Results					
	λ_h_	e_0_	$R_{b,n}^{\exp}$, MPa	$N_{}^{\exp},$ kN	*x*, cm		$N_{cr}^{},$ kN	$\eta^{theor}$	$N_{}^{theor},$ kN	$\frac{N_{}^{theor}}{N_{}^{\exp}}$
1	2	3	4	5	6	7	8	9	10	11
AKY-X_1_	10	0.2	282.3	1190.5	11.1		518,800	1.28	1156	0.97
AKY-X_4_	10	0.2	363.7	1600	11.3		797,093	1.22	1462	0.91
AKY-X_5_	10	0.2	283.0	1625	11.2		976,040	1.15	1275	0.78
BKY-X_1_	10	2.2	282.3	778.9	7.3		433,565	1.18	676	0.87
BKY-X_2_	10	2.2	284.5	794.7	7.3		443,950	1.18	699	0.88
BKY-X_5_	10	2.2	302.9	844	7.3		473,548	1.19	777	0.92
CKY-X_1_	10	4.2	309.9	482.5	5.17		318,305	1.16	450	0.93
ΣΔ^2^ = 0.06										

## Data Availability

The study did not report any data.
